# SALGOT - Stroke Arm Longitudinal study at the University of Gothenburg, prospective cohort study protocol

**DOI:** 10.1186/1471-2377-11-56

**Published:** 2011-05-25

**Authors:** Margit Alt Murphy, Hanna C Persson, Anna Danielsson, Jurgen Broeren, Åsa Lundgren-Nilsson, Katharina S Sunnerhagen

**Affiliations:** 1 Department of Clinical Neuroscience and Rehabilitation, Institute of Neuroscience and Physiology, Sahlgrenska Academy at University of Gothenburg, Gothenburg, Sweden

**Keywords:** stroke, upper extremity, recovery of function, kinematics, longitudinal study

## Abstract

**Background:**

Recovery patterns of upper extremity motor function have been described in several longitudinal studies, but most of these studies have had selected samples, short follow up times or insufficient outcomes on motor function. The general understanding is that improvements in upper extremity occur mainly during the first month after the stroke incident and little if any, significant recovery can be gained after 3-6 months. The purpose of this study is to describe the recovery of upper extremity function longitudinally in a non-selected sample initially admitted to a stroke unit with first ever stroke, living in Gothenburg urban area.

**Methods/Design:**

A sample of 120 participants with a first-ever stroke and impaired upper extremity function will be consecutively included from an acute stroke unit and followed longitudinally for one year. Assessments are performed at eight occasions: at day 3 and 10, week 3, 4 and 6, month 3, 6 and 12 after onset of stroke. The primary clinical outcome measures are Action Research Arm Test and Fugl-Meyer Assessment for Upper Extremity. As additional measures, two new computer based objective methods with kinematic analysis of arm movements are used. The ABILHAND questionnaire of manual ability, Stroke Impact Scale, grip strength, spasticity, pain, passive range of motion and cognitive function will be assessed as well. At one year follow up, two patient reported outcomes, Impact on Participation and Autonomy and EuroQol Quality of Life Scale, will be added to cover the status of participation and aspects of health related quality of life.

**Discussion:**

This study comprises a non-selected population with first ever stroke and impaired arm function. Measurements are performed both using traditional clinical assessments as well as computer based measurement systems providing objective kinematic data. The ICF classification of functioning, disability and health is used as framework for the selection of assessment measures. The study design with several repeated measurements on motor function will give us more confident information about the recovery patterns after stroke. This knowledge is essential both for optimizing rehabilitation planning as well as providing important information to the patient about the recovery perspectives.

**Trial registration:**

ClinicalTrials.gov: NCT01115348

## Background

Stroke is defined by the World Health Organization (WHO) as rapidly developing clinical signs of focal or global disturbance of cerebral function, with symptoms lasting more than 24 hours or leading to death and with no apparent non-vascular cause. The incidence of stroke in Sweden is 300 cases per 100 000 inhabitants in a year of whom 200 suffer a first incidence of stroke leading to a total of 18 000 new stroke victims. About 25000 - 30000 persons yearly suffer from acute stroke each year in Sweden. Of these, about 20% will die within the first month and about 1/3 of the survivors will remain significantly disabled after 6-12 months [1].

The upper extremity function is impaired after stroke in approximately 70-80% of patients in acute phase and in 40% in chronic phase [2-4]. This impairment limits the voluntary, well coordinated, and effective movements as well as a person's level of activity [5] and participation in their social and physical environment [2]. This longstanding disability might also influence the quality of life [6].

Recovery of motor skills after stroke depends both on spontaneous reparative process as well as reorganization of neural mechanisms, influenced by inputs and demands given to the motor control system. The current perspective on motor learning focuses on active task-oriented training and how feedback and other basic training principals such as regularity, intensity and specificity affects the long-term recovery [7,8]. In order to detect meaningful improvements in motor function, appropriate outcome measures should be used. Beside the requirements on reliability, validity and sensitivity, the issues of functionality and objectivity must be considered while selecting the appropriate measures. Assessment methods with continuous variables are recommended to be included into evaluation batteries since they might have higher power to detect the important improvements in motor recovery [9-11].

Improved understanding of the recovery patterns after stroke is essential for planning and execution of optimal rehabilitation. Recovery patterns of upper extremity function have been described for selected stroke populations in several longitudinal studies. The general idea is that improvements in the upper extremity occur mainly during the first month after onset of the stroke and that little, if any, significant recovery can be gained after 3-6 months [3,12-14]. Several studies, conducted in selected populations at rehabilitation facilities have shown that, in some patients, the improvements also continued for a longer time [2,4,15]. There are only a few studies with non-selected community based populations describing the recovery patterns in the upper extremity. These studies report a similar recovery pattern with little or no significant recovery beyond 2-3 months [3,16-18]. Whether this is correct is not clear for the non-selected studies, since in some reports the sample sizes were small [14,15], the follow up times were short [3,4] or the information on the motor assessments was not satisfactory [3,18].

### Kinematic measurement - drinking task

Kinematics describes movements of the body through space and time, including linear and angular displacements, velocities and accelerations, but without reference to the forces involved. Kinematic data can be achieved by optoelectronic systems where multiple high-speed cameras send out infra red light signals and detect the reflection from the markers placed on the body. Kinematic variables provide objective, precise and detailed measures of movement performance and quality.

Kinematic movement analysis has become a useful assessment tool within rehabilitation and is employed routinely for gait analyses. Few studies have used kinematic movement analysis to examine the upper extremity in a longitudinal design. In one of these studies the kinematic data was obtained from an isolated fast elbow extension [15,19] and in the other a targeting fast reaching movement [20]. In order to better understand the situation of a person with impaired upper extremity function, information is needed regarding activities of daily living. It is known that the motor activity of the upper extremity is dependent on the meaning of the task and on the shape and placement of the object [21]. Thus, it is meaningful to study natural purposeful movements with real-life objects. In an earlier study we have developed a test protocol and a program for data analyses of the kinematic variables for the activity of drinking from a glass, which has been applied in a control setting [22] and in stroke subjects [23].

### Kinematic measurement - Virtual reality test

Virtual reality (VR) can be described as the world perceived in a computer. VR systems that include a haptic device can provide tactile feedback to the user through the force feedback. If the system detects a collision between the device and virtual objects, it transmits a reaction to the user's hand, which interacts with perception of the test or training situation [24]. In the real world, objects are usually perceived in the same location whether the sense involved is vision or touch (haptic). In the virtual world, the precise co-location of haptics is technically harder to achieve, but when the co-location is accurate the realism of the manipulation is very high and the user's performance is improved [25]. The knowledge about effects of using VR in assessments and training after stroke is still limited, but sufficiently encouraging to justify additional clinical trials in this population [26-31].

### Theoretical background

WHO approved in May 2001 the model on International Classification of Functioning, Disability and Health (ICF) [32] to assess the consequences of a disorder or a disease on the individual person. The ICF model provides a multi-perspective approach to the classification of functioning and disability as an interactive and evolutionary process. In the model an individual's functions in a specific domain is an interaction or complex relationship between the health conditions (physical or mental) and contextual factors (social and physical environment as well as personal factors). The components of ICF can be used to indicate problems (e.g. impairments, activity limitations or participation restrictions summarized under the umbrella term disability) in different areas. This approach forces health professionals to look wider than the usual perspective, which has traditionally lain in the domain of body function and structures. The model boosts the traditional rehabilitation ideology where the focus has not been on the organ but on the person and thereby requiring different treatments depending on that person's goal. In order to assess the consequences of a disease we need to look at different components of the ICF.

Longitudinal studies are difficult to perform. Sweden has a unique situation since people are quite easy to trace through the civic system and moving from one region to another is not so frequent. In addition, the representativeness for the disease is good since all patients within a catchment area are usually referred to the same hospital as private alternatives are scarce and thereby the possibilities to generalize the results are good.

The purpose of this study is to describe the recovery of upper extremity function longitudinally in a non-selected sample with first ever clinical stroke admitted to a stroke unit.

The specific objectives of the present study are to:

A. Follow recovery of upper extremity by using clinical measures of body function (motor function, spasticity), activity (use of the arm and hand) and participation (impact of limitations) after stroke

B. Follow functional recovery by using objective, new IT technology (kinematic movement analysis and VR-test with sensory feedback) after stroke

C. To gather the assessments of participants self-perceived upper extremity function over the first year after stroke

D. To predict function at 12 months by analysis of data gathered at first week after onset of stroke

## Methods/Design

A sample of 120 persons with a first occurrence of stroke will be included and followed longitudinally for one year after the stroke. The group will consist of consecutively included persons recruited from the stroke unit at Sahlgrenska University Hospital, Gothenburg, Sweden. The Stroke unit at Sahlgrenska University Hospital serves the larger Gothenburg urban area, thus all persons from this catchment area are randomly referred to the Sahlgrenska University Hospital. The project is approved by the Regional Ethical Review Board and the Helsinki declaration is followed. Written informed consent will be obtained from the participants or from their closest relative. The SALGOT study is registered on ClinicalTrials.gov (NCT01115348).

Inclusion criteria are:

• Diagnosed first ever clinical stroke, based on WHO criteria (ischemic infarct, haemorrhagic and subarachnoidal bleeding)

• Impaired upper extremity function. This is defined in two steps. On the first or second day after stroke onset the upper extremity function is assessed with Modified Motor Assessment Scale (M-MAS UAS-95) [33] (this is performed as standard clinical assessment by physiotherapists working at the stroke unit). All persons, who do not obtain the maximum score on the subtests of arm function, hand movements and fine motor function due to hemiparesis, will be informed about the study and retested at day three after stroke with Action Research Arm Test (ARAT) [34]. All persons who do not achieve the maximum score for ARAT (score 57) will be included.

• Admitted to the stroke unit within three days after stroke onset

• Living in the Gothenburg urban area (maximal 35 km from the Sahlgrenska University Hospital)

• Age 18 or older

Exclusion criteria are:

• Upper-extremity injury or condition prior to the stroke that limits the functional use of the affected arm and hand

• Severe multi-impairment or diminished physical condition before the stroke that will affect the arm function

• Life expectancy less than 12 months due to other illness (cardiac disease, malignancy) or severity of stroke injury

• Not Swedish speaking prior to the stroke incident

### Design and procedure

**Figure 1:**
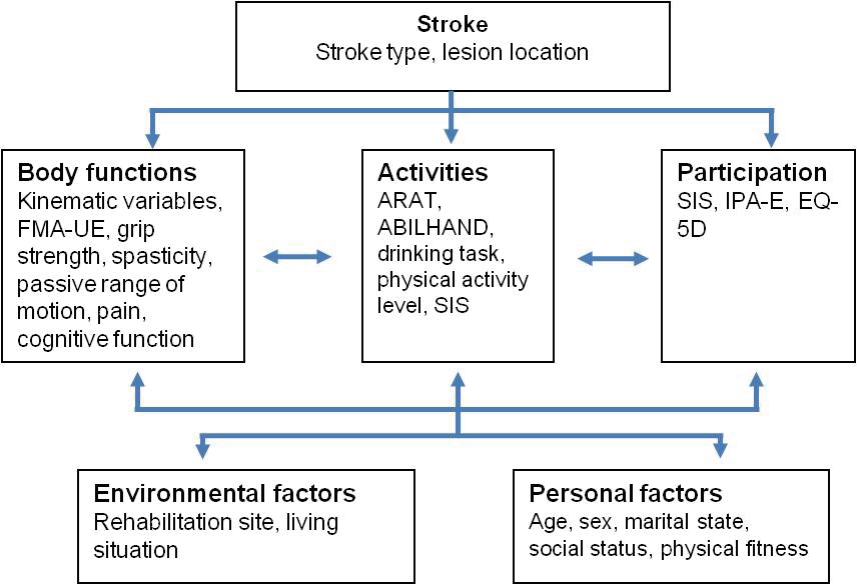
**Outcome measures used in SALGOT study according to ICF classification**.

This study will evaluate the recovery patterns after first ever stroke without any intervention except standard rehabilitation planning and procedures. All included participants will be assessed eight times during the first year after stroke. Assessments are performed at day 3 and 10, week 3, 4 and 6, month 3, 6 and 12 after onset of stroke. Tests are administrated in block randomized manner in order to minimize the systematic testing bias. The test order and the reason for missed or unsuccessful test results will be recorded in a protocol. All tests are performed by three experienced physical therapists, undergoing a training period together for the assessment battery prior to the study start. ICF classification of functioning, disability and health is used as framework for the selection of assessment measures (Figure 1).

### Outcome measures

Demographic data will be collected during the first assessment. Stroke subtype will be confirmed by CT and/or MRI scans. Ischemic strokes will be classified for subtype and site for lesion by using TOAST [35] and Bamford classifications [36]. Treatments of thrombolysis or thromboectomy will be registered. Additional data will be extracted from the national quality register for stroke - Swedish Stroke Register [1]. The Self-Administrated Comorbidity Questionnaire (SCQ) will be used to collect additional information on relevant medical conditions and problems [37]. Cognitive function is evaluated at every test occasion using Barrow Neurological Institute Screen for Higher Cerebral Functions (BNIS) [38]. The three prescreen items scoring the level of consciousness/alertness, cooperation and basic communication skills and the item of auditory comprehension will be assessed. The level of physical activity is recorded by a 6-grade scale of Physical Activity Classification [39,40]. This instrument is valid, short and suitable for longitudinal studies and takes account the activity level both during domestic and fitness activities [40]. Exact time points for all assessments are listed in Table 1.

**Table 1:** Scheme over the assessments and time-points for test occasions

Assessments	Test occasion (d=day, w=week, m=month)								
	**d1**	**d3**	**d10**	**w3**	**w4**	**w6**	**m3**	**m6**	**m12**

**M-MAS UAS -95**	x								

**NIHSS**	x								

**BNIS**		x	x	x	x	x	x	x	x

**Physical activity scale**		x						x	x

**FMA-UE**		x	x	x	x	x	x	x	x

**Action Research Arm Test**		x	x	x	x	x	x	x	x

**ABILHAND**		x	x	x	x	x	x	x	x

**Grip strength**		x	x	x	x	x	x	x	x

**Modified Ashworth Scale**	x	x	x	x	x	x	x	x	x

**Kinematic - drinking task**		x	x		x		x	x	x

**Kinematic - VR-test**		x	x	x	x	x	x	x	x

**Stroke Impact Scale**			x		x		x	x	x

**IPA-E**									x

**EQ-5D**									x

### Clinical outcome measures of function and activity

The upper extremity motor function will be assessed using the Fugl-Meyer Assessment for Upper Extremity (FMA-UE) [41], and a maximum score of 66 corresponds to normal motor function. The psychometric properties of Fugl-Meyer Assessment have shown excellent reliability and validity [41-43]. The non-motor domains of FMA-UE, sensation, passive range of motion and pain during passive joint motions will be completed as well.

Action research Arm Test (ARAT) is a performance test for upper extremity function and dexterity [44]. The ARAT uses ordinal scoring on 19 items divided into four hierarchical subtests: grasp, grip, pinch and gross movement. Each upper extremity is evaluated individually and the test can be completed in 5-15 minutes [44,45]. ARAT has been shown to have good validity, sensitivity to spontaneous and therapy-related gains after stroke both in acute and chronic phase [44,46]. The ARAT has shown good responsiveness [47] and excellent inter-rater and intra-rater reliability [44,48].

Spasticity will be assessed with the Modified Ashworth Scale (MAS). The muscle groups of elbow flexors and extensors, wrist flexors and extensors will be evaluated. The MAS is the best alternative for spasticity assessment in clinical setting available and has been shown to have fair reliability for these joints [49,50].

The grip strength will be measured using the Jamar Hand Dynamometer. Standardized positioning and instructions are followed and the average of three trials is used as test outcome [51]. Reliability for the grip strength measure is very high [52].

### Kinematic measurements - objective outcomes of performance

**Figure 2:**
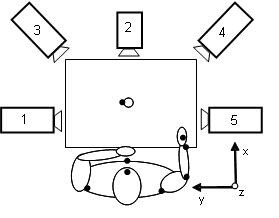
**Setup of kinematic 5-camera motion capture system for the drinking task**. Participant is presented with the right arm in initial position, and marker sites are shown as black dots.

Three-dimensional motion analysis of upper extremity during drinking task will be performed with a 5-camera optoelectronic ProReflex Motion Capture System (MCU240 Hz, Qualisys AB, Sweden). The tracing of the three-dimensional coordinate positions of the markers is completed automatically by Qualisys Track Manager, 2.0. The capture data is then transferred to MATLAB (The MathWorks Inc) software for custom-made analysis. A standardized drinking task with stable test-retest reliability will be used [53]. The participant is sitting in front of the table with tested hand resting on the edge of the table (Figure 2). A drinking glass, filled with 100 mL water is placed 30 cm from the table edge in the midline of the body. The drinking task includes reaching, grasping, and lifting the glass from the table and taking a drink (one sip); placing the glass back on the table behind a marked line; and returning to the initial position. Participants are instructed to sit against the chair back during the whole task, but the sitting position is not restrained, and compensatory movements are allowed. All participants perform the drinking task at a comfortable self-paced speed, starting with their non-affected arm, after practicing a few times. The mean of the three middle trials of total five will be used for statistical calculations. A total of 9 spherical 12-mm retroreflective markers are placed on the third metacarpophalangeal joint of hand, styloid process of ulna on wrist, lateral epicondyle of elbow, middle part of acromion on right and left shoulder, upper part of sternum, forehead and on the upper and lower edge of the glass. The procedure has been described in more detail previously [53,54].

**Figure 3:**
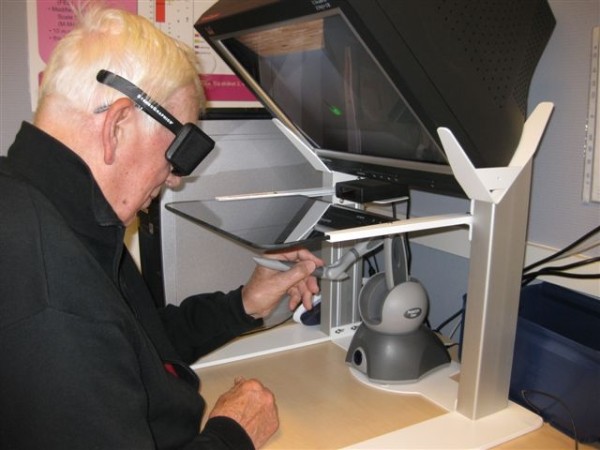
**Participant is performing the VR-test**. The VR equipment consists of a semi-immersive workbench with haptic device and stereoscopic glasses.

In the VR test [55], the participant reaches into a virtual space and interacts with 3D objects. The VR equipment consists of a semi-immersive workbench with haptic device and stereoscopic glasses. In our set-up, the haptic equipment looks like a stylus shaped instrument attached to a lever system and it is freely movable in all directions (Figure 3). During the test, the position of the stylus is tracked, and resistive force is applied to the stylus when it comes into contact with the virtual object, providing force feedback. In addition to the visual perception, the haptic device creates an illusion of manipulation and sensation of the virtual objects. The participant moves the stylus in a realistic environment, experiencing the sense of moving inside the computer screen. The precise co-location of haptics is achieved by projecting the virtual image onto the same location as the user's hand through the mirror setup. The VR-test, developed by our group, is a precise quantitative kinematic measurement tool for arm and hand movements and has been shown to have a good test retest reliability [31,56,57]. During the test the participant has to move the haptic stylus to 32 different targets in the virtual environment (VE) generated by the computer. The targets appear one after the other and disappear when touched. Each target consists of a whole circle (diameter 3.0 cm viewing angle). The 32 target placements in the VE are random to the subject but are actually set according to a pre-set kinematic scheme for evaluation purposes. In each test occasion the participant have one or two training trails before the measurements starts. Both dominant and non-dominant hand is measured, starting with the non-dominant hand. The participant performs the test as fast as possible.

### Self-perceived outcomes

ABILHAND [58,59] is a questionnaire aiming to assess manual ability in persons with chronic stroke. It is interview based and focused on perceived difficulties in everyday activities. A Swedish version has been validated [60]. ABILHAND is a Rasch-based assessment; it is unidimensional and can be used as linear measure [58,59].

Stroke Impact Scale (SIS) [61] is a questionnaire on different aspects of the stroke recovery where the person replies on their perception regarding their life after the stroke. The 59 questions are divided into 8 domains; strength, memory, emotion, communication, activities of daily living, mobility, hand function and social participation. Items within the domain are ordered hierarchically based on clinical perspective and Rasch analysis [62]. Only the first four sections are used for the test occasion at day 10.

Impact on Participation and Autonomy (IPA-E) is a generic outcome measure for adults with chronic conditions where the person estimates perceived limitations in participation and autonomy related to dependency in the current living surrounding [63-65]. The subscales include autonomy indoors, family role, autonomy outdoors, social life and relationships, work and education. Additionally, IPA-E identifies the extent to which limitations in life are experienced as problematic in areas of mobility, self care, activities, economy issues, social life, work and education. IPA-E is valid, reliable and sensitive to change after stroke [63-65].

EuroQol Quality of Life Scale (EQ-5D) will be used to measure the health status related to the quality of life. It is a widely used generic measure and includes five dimensions: mobility, self-care, usual activities, pain/discomfort and anxiety/depression [66,67].

### Data analysis

The kinematic data in the drinking task is filtered with a 6-Hz second-order Butterworth filter, resulting in zero-phase distortion and fourth-order filtering. The drinking task is broken down into five logical phases: reaching for the glass, forward transport of the glass to the mouth, drinking, back transport of the glass to the table, and returning the hand to the initial position. The selection of kinematic variables and data analysis calculations will be based on our earlier studies [53,54]. Movement onset is defined as the time when the tangential velocity of the hand marker exceeds 2% of the maximum velocity in the reaching phase. Movement offset is detected when the velocity of the hand is less than 2% of the maximum velocity in the returning phase. Start of forward transport phase is defined as the time when the tangential velocity of the glass exceeds 15 mm/s. The drinking phase is identified by a 15% increase or decrease of the steady-state distance between the face and glass marker. The start of the returning phase is defined as the time when the tangential velocity of the glass is less than 10 mm/s. Movement times are calculated for the whole movement and separately for each phase. Peak tangential velocity and angular velocity of the elbow joint are computed for the reaching phase. Smoothness of movement is quantified by computing the number of movement units during the reaching and forward transport phases [53]. Angular joint motions are computed from the 3D position data for elbow flexion/extension, shoulder flexion/extension in the sagittal plane, and abduction/adduction in the frontal plane [53]. Compensatory trunk movement is computed for the entire drinking task as the maximal displacement of the thorax marker from the initial position [53]. Interjoint coordination between the shoulder and elbow joint angles for reaching phase is computed using cross-correlation analysis of zero time lag [53].

In the VR-test hand position data (haptic stylus end-point) will be gathered. The position of the stylus is tracked and resistive force is applied to it when it comes into contact with the virtual model, providing force feedback. All measurements generate time-stamped motion data (x, y, z) at 1000 Hz. Different parameters such as reaction- and movement time, velocity, acceleration and deceleration times are calculated. To obtain the movement quality of the hand trajectory, a hand path ratio, corresponding to the length of the pathway is calculated. The selection of kinematic variables and data analysis calculations will be based on our earlier study [30].

The raw scores from the ABILHAND questionnaire are analyzed using a Rasch analysis computer program and expressed as logistically transformed probability measures, logits [68]. In the Rasch model the raw scores are used to estimate the linear ability for each subject and linear difficulty for each item of measurement around a unidimensional continuum. Thus, the Rasch model converts the ordinal score of subject's manual ability into an equal interval linear measure.

### Group size/power analysis

Prior longitudinal studies stroke cohorts at Sahlgrenska University Hospital have had a dropout rate of 30%. With a power (1-β) at 0.8 and a significance level (α) at 0.05, we need a sample of 88 patients (two-sided test) to determine a medium effect of 6 points change (10%) on ARAT. Therefore, we aim to include 120 persons.

## Discussion

The SALGOT study is a longitudinal prospective study with a non-selected sample from Gothenburg urban area. A sample of 120 persons with first ever clinical stroke admitted to a stroke unit will be consecutively recruited from Sahlgrenska University Hospital. The study is non-interventional and the main goal is to describe the recovery of upper extremity function after first ever clinical stroke and to follow the improvements and consequences of stroke during the first year in these persons life. Measurements are performed both using traditional clinical assessments as well as computer based measurement systems that provide objective kinematic data. The person's perspective of recovery is captured both with stroke specific as well as generic self-perceived outcome measures.

In this study, the participants are assessed at eight occasions during the first year after stroke. This design gives an opportunity to study which persons will recover, when and in which areas the recovery occurs. From earlier studies it is known that the improvement of function is mostly gained during the first months after stroke. But the majority of these reports have been conducted on selected populations and in many studies the selection of outcome measures on motor function has not been sufficient. Additionally, new technologies obtaining objective kinematic measures on motor function and performance have been scarcely used in longitudinal studies.

The gained knowledge of recovery patterns is necessary both for the healthcare system and for the individual who has suffered a stroke. Since the rehabilitation resources are limited, there is a need to know the optimal time point for interventions and have guidelines for rehabilitation planning. The more detailed information about the recovery patterns of upper extremity is needed in order to offer individualized assessment and treatment, to inform the patient sufficiently about the recovery perspectives and to enhance the patient's motivation for the rehabilitation period.

## Abbreviations

ARAT: Action research Arm Test; BNIS: Barrow Neurological Institute Screen for Higher Cerebral Functions; EQ-5D: EuroQol Quality of Life Scale; FMA-UE: Fugl-Meyer Assessment for Upper Extremity; IPA-E: Impact on Participation and Autonomy; M-MAS UAS-95: Modified Motor Assessment Scale accordingly Uppsala Akademiska Sjukhus 95; NIHSS: National Institutes of Health Stroke Scale; SIS: Stroke Impact Scale; TOAST: Trail of Org 10172 in Acute Treatment; VR: Virtual reality; VE: Virtual Environment.

## Competing interests

The authors declare no competing interests.

## Authors' contributions

MAM and HCP participated in the conception and design, planning, managing the process and are responsible for day-to-day management of the study. KSS initiated the study, participated in the conception and design, managed the process and drafted the initial manuscript. All authors contributed to the study planning, drafting the manuscript and have approved the final manuscript.

## Pre-publication history

The pre-publication history for this paper can be accessed here:

http://www.biomedcentral.com/1471-2377/11/56/prepub
